# Artificial intelligence-based grading of human cataracts: A feasibility study

**DOI:** 10.1371/journal.pone.0355969

**Published:** 2026-08-13

**Authors:** Catharina Latz, Franziska Rothen, Annika Licht, Katharina A. Ponto, Alireza Mirshahi

**Affiliations:** 1 Department of Ophthalmology, Marien Hospital, Düsseldorf, Germany; 2 University of Bern, Biomedical Engineering, Bern, Switzerland; 3 Dardenne Eye Hospital, Bonn, Germany; 4 Department of Ophthalmology, University Medical Center, Mainz, Germany; PLOS ONE, UNITED KINGDOM OF GREAT BRITAIN AND NORTHERN IRELAND

## Abstract

This feasibility study developed and validated an AI algorithm for automatic classification of human lens opacification (cataracts) using anterior segment optical coherence tomography (OCT). The dataset comprised 1,802 unprocessed OCT images from 901 eyes, with cataract severity graded by slit-lamp examination using the Lens Opacities Classification System (LOCS) III. Nuclear opalescence (NO) was stratified as mild (≤3.0), moderate (3.5–4.0), or severe (≥4.5), while posterior subcapsular cataract (PSC) was classified as mild (<4) or severe (≥4). Nuclear color (NC) and cortical cataract (C) were not evaluated. After balancing, images were randomly divided into three sets: 70% for training, 20% for testing and 10% for validation. A convolutional neural network was trained five times. Patients had a mean age of 72 ± 8.8 years, with mean LOCS III grades of 3.81 ± 0.79 (NO) and 1.74 ± 1.55 (PSC). The AI achieved 60.3% accuracy for NO classification; among misclassifications, 34.9% were within one category, 4.8% were grossly inaccurate. PSC classification performed with 86.4% accuracy, yielding a true positive rate of 81.8% and a true negative rate of 90.9%. These results demonstrate the potential of deep learning for automated cataract grading.

## Introduction

Cataract is the leading cause of reversible vision loss and blindness worldwide and cataract surgery, the only permanent treatment option, is one of the most performed surgical procedures in the developed world [1,2]. Femtosecond laser-assisted cataract surgery (FLACS) is an established variant of this procedure offering additional precision and safety [3]. The Lens Opacities Classification System III (LOCS III) is a widely established patient-derived subjective grading method for cataracts and lens hardness [4]. Using a set of standard slit-lamp photographs and retroillumination images as a reference, graders assess cataract status based on four different characteristics: nuclear colour, nuclear opalescence, cortical cataract and posterior subcapsular cataract. As this is a subjective, time-consuming and costly process, artificial intelligence (AI)-based applications have been developed in recent years mostly based on slit lamp and fundus images to enable objective and automated classification [5].

Nowadays, optical coherence tomography (OCT) images are also routinely used in the diagnosis of anterior segment eye diseases [6]. In addition, most femtolaser platforms utilize OCT imaging to verify the position of intraocular structures, such as the iris and the crystalline lens. As cross-sectional images of the anterior segment of the eye, they provide objective and quantitative information about the cornea and lens. This enables detailed in-depth information and allows measurement of lens density. Lens density directly influences the amount of energy required to fragment the cataractous lens during surgery. A correlation between the LOCS III nuclear opalescence (NO) grade and nuclear density measured on OCT images has been demonstrated in several studies [7–10].

Although De Castro et al. demonstrated that clinically observed lens changes correspond to hyper- and hyporeflective patterns on swept-source (SS) OCT images [11], grading cataracts remains challenging. The homogeneous and continuous grayscale variations seen on OCT images with nuclear cataracts are difficult to assess accurately with the naked eye. Therefore, image processing and feature extracttion are often applied.[7,12,13]

Although some machine-based grading systems have achieved high accuracy, they require feature extraction from up to 64 OCT images. In addition, these approaches evaluated only nuclear color and used a simplified grading system limited to three categories: no, mild, and severe nuclear cataract [5,11,14,15]

The aim of this feasibility study was to develop and validate an AI-algorithm for the automatic classification of nuclear cataract and posterior subcapsular cataract (PSC) using only two unmodified OCT images of the human lens. OCT images were acquired intraoperatively with the Femtolaser (Ziemer FEMTO LDV Z8). To establish a connection between the lens density determined by OCT and LOCS III NO and PSC grades, SD-OCT images of the anterior segment of patients with known LOCS III grading were used as a database.

## Methods

This feasibility study was conducted from January first, 2022 to February first, 2023 in collaboration with the Dardenne Eye Clinic (Bonn) and the University of Bern. The study included SD-OCT images of the anterior segment from patients with age-related as well as secondary cataract and known LOCS III grading. Traumatic, congenital and pediatric cataracts were excluded. While retrieving LOCS III grading information, AL had access to information that could potentially identify individual participants during or after data collection. Through written consent to the privacy policy, patients were informed about the conduct of medical research and their right to object to the use of their data for research purposes. All data were fully anonymized before being accessed outside of the Dardenne Clinic. The ethics committee of the Rhineland Medical Chamber waived the requirement for additional informed consent and stated that ethical approval is not required for retrospective analysis of anonymized datasets.

At the Dardenne Eye Clinic, LOCS III gradings of the human lens are routinely performed for all cataract patients prior to surgery. The grading process is conducted based on reference images by two highly experienced ophthalmologists who are regularly trained in the LOCS III grading system. OCT images are automatically acquired intraoperatively from all patients undergoing femtosecond laser-assisted cataract surgery, as the image acquisition serves as location control for the laser energy. Our clinic uses the Ziemer FEMTO LDV Z8 with a spectral domain OCT system, which is highly specialized for corneal and lens surgery. This system captures two intraoperative SD-OCT images of the anterior segment of the eye at a 90-degree angle to each other after laser docking. Because a minimum diameter of 5.0 is required to safely perform the laser capsulotomy without risking damage to the iris, only eyes with an intraoperative pupil diameter of > 5.0 mm were included into the study.

### Dataset, AI architecture, and training

From a clinical perspective, a cataract classification into three categories (mild, moderate and severe) is adequate to indicate the need for surgery, or to adjust settings of the femtosecond laser or the phacoemulsification system if required. To train the AI-algorithm for nuclear cataract, the known LOCS III NO grades assigned to the OCT images were therefore categorized as follows: ‘mild’ corresponds to LOCS III grades ≤ 3.0, ‘moderate’ includes LOCS III grades 3.5 and 4.0, while ‘severe’ includes LOCS III grades ≥ 4.5. Due to the limited quantity of training data for PSC, especially in the higher grades, a binary classification model was used, classifying LOCS III PSC grades below 4 as ‘mild’ and scores of 4 or more as ‘severe’.

A total of 1802 OCT images from 901 eyes with age related or secondary cataract and known LOCS III grading served as the image database (Table 1). Mean age of patients was 72 ± 8.76 years (range: 26–92 years).

**Table 1:** Patient demographics.

Parameter	Range	Mean + /- SD
Patients (n)		618
OCT scans (n)		1802
Age* (years)	26-92	72.31 + /- 8.76
LOCS III NO grade	2.0-6.0	3.81 + /- 0.70
LOCS III PSC grade	0.0-6.0	1.74 + /- 1.55

* at the time of surgery.

To avoid uneven data distribution, which could lead to a biased network due to distorted input data, a balanced dataset with an equal amount of OCT image data per category was used for training. To achieve this, firstly, all the OCT images with a known LOCS NO grading were categorized into mild, moderate and severe. Then, the amount of OCT images in each category was adjusted to be equal to that of the category with the least images. The same procedure described for the two categories was applied to OCT images with LOCS III PSC grading.

**Fig 1:**
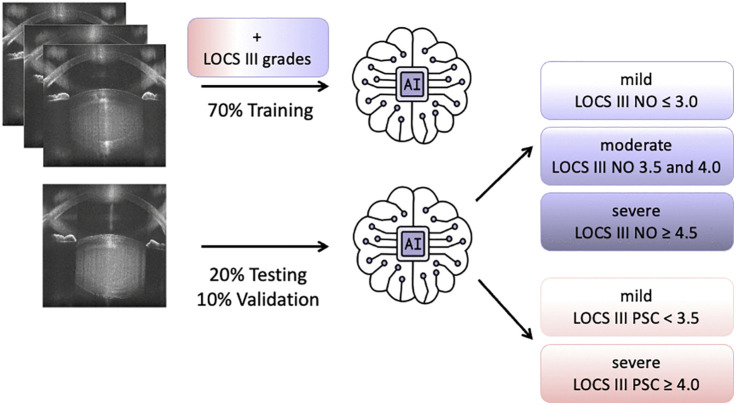
AI approach. The convolutional neural network was trained (upper section) to automatically classify lens density of nuclear cataracts from OCT-images into three NO or two PSC categories (lower section of figure).

After balancing the dataset, 1254 and 428 OCT images with LOCS NO and LOCS PSC grades respectively were randomly assigned to training (70%), test (20%) and validation set (10%). The pretrained EfficientNet-B0, a convolutional neural network (CNN)-based AI algorithm, was trained to classify the cataract grade from full anterior segment OCT images using this database (Fig 1). To enhance result reliability, the network was trained five times and the final prediction of the AI model for each OCT image was determined using the soft majority vote (predicting a class by choosing the highest summed probability).

### Statistical analysis

To assess the performance of the AI-based classification model, confusion matrices were created, showing the predicted vs. true LOCS III grades. The classification accuracy for both models was calculated based on the determined numbers of True Positives (TP, i.e., the sample is predicted as positive and is actually positive), True Negatives (TN, i.e., the sample is predicted as negative and is actually negative), False Negatives (FN, the sample is predicted as negative but is actually positive), and False Positives (FP, the sample is predicted as positive but is actually negative).

For the 3-class grading model for nuclear cataracts, the accuracy was additionally determined by calculating the Cohen quadratic kappa score based on the multiclass confusion matrix [16].

Gradient Weighted Class Activation Maps (Grad-CAM) were utilized to get an insight into how the AI-based classification model was operating and which regions of an image were relevant for classification [17]. These were displayed in a heat map.

## Results

**Fig 2:**
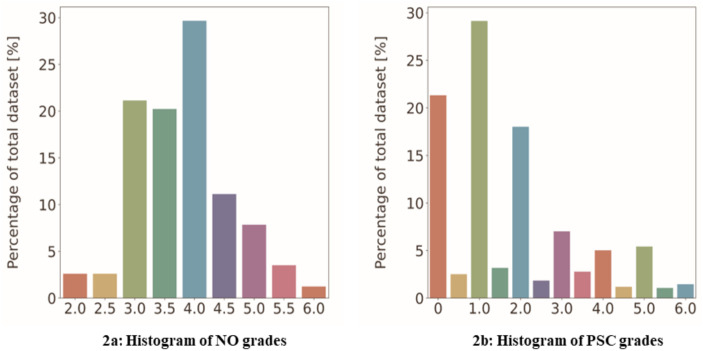
Histogram of NO and PSC cataract distribution of complete dataset.

The distribution of LOCS III NO grades in the underlying dataset (n = 1760) was centered on medium severity, with most patients having a cataract of LOCS III NO grade 3–4 (Fig 2a). As this dataset only includes cataracts requiring surgery, LOCS III NO grades of 2.5 and lower are rare. The distribution of LOCS III PSC grades of the dataset (n = 1510) is shown in Fig 2b. Since PSC cataract is by definition centered on the central 3 mm of the visual axis it becomes visually significant at an early stage. This is seen in the high proportion of low PSC grades.

### Classification of nuclear cataract

**Fig 3:**
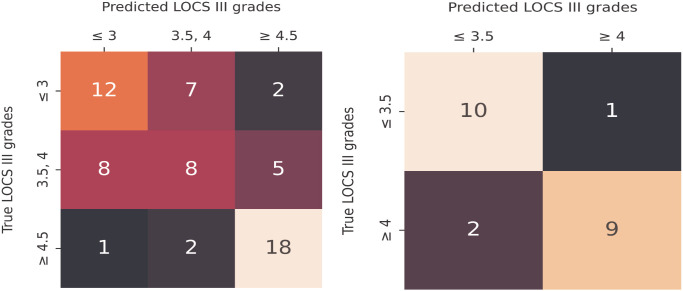
Confusion matrix. (A) NO classification, (B) PSC classification.

Fig 3a shows the confusion matrix for the 3-class model for nuclear cataract classification (mild, moderate, severe). The calculated accuracy of the model in the validation set is 60.3% (N = 63). When using a 3-class model, it is important to consider that an incorrect prediction by one category is less relevant than an incorrect prediction by two categories. To address this, incorrect predictions were classified as either ‘near miss’ or ‘worst case predictions’. The AI-based model’s ‘near misses’, where the prediction was off by one category (e.g., mild instead of moderate), accounted for 34.9% of cases. ‘Worst case predictions’, where the prediction was off by two categories, accounted for only 4.8% of cases. The Cohen quadratic kappa score was 0.614.

### Classification of PSC

The confusion matrix for the binary classification system for categorizing PSC into ‘mild’ (LOCSIII ≤ 3.5) and ‘severe cases’ (≥4) is shown in Fig 3b. In the validation set, the resulting accuracy is 86.4% (N = 22), true positive rate (sensitivity) 81.84% and true negative rate (specificity) 90.9%.

### Class activation map

**Fig 4:**
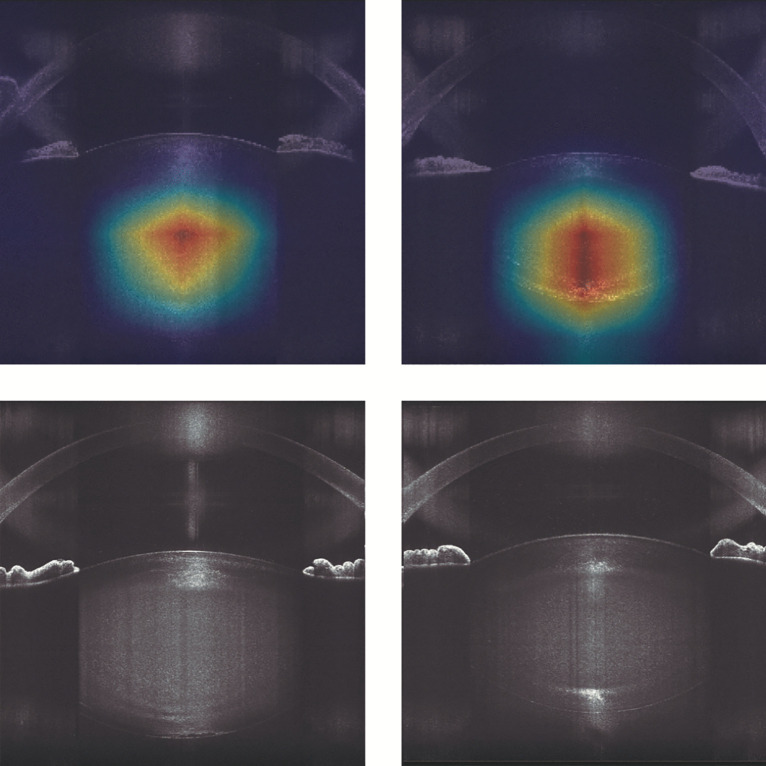
Heatmap of grad-CAM images. Overlay of all OCT images from the validation set, (A) NO, (B) PSC, (C) example OCT image of NO grade 6, (D) example OCT image of PSC grade 6.

Grad-CAM were used to visualize, which parts of the image are most important for AI-based classification by using a heat map. The results show that the trained network is correctly focused on the crystalline lens, while the regions around the lens have a relatively small influence on the classification (Fig 4). For PSC grading the posterior region of the lens is hotter than for nuclear grading.

## Discussion

The feasibility study results indicate that an AI-based approach, utilizing a deep learning algorithm based on a convolutional neural network (CNN), can automatically classify nuclear cataract lens density based on OCT images, confirming previous findings [5,14].

The CNN-based model developed in this project can classify both nuclear cataract and PSC with a reasonable degree of accuracy and can distinguish between mild and severe cases. In contrast to other approaches, pixel intensity analysis for example [9], this AI model assesses features such as structures and patterns within the crystalline lens. This contributes to a more comprehensive image evaluation, potentially resulting in higher accuracy. In 2008, Wong et al. showed a correlation of r = 0.77 between the NO LOCS III score and the nuclear density evaluation based on AS OCT obtained with the Visante OCT Model 1000 (Carl Zeiss Meditec) [8]. For this study, the OCT-images had to be exported and analyzed with an external image analysis software. In addition, the background noise was mathematically subtracted. In comparison, Mackenbrock et al published a study in 2022, where OCTs obtained with an SS-OCT (ANTERION, Heidelberg Engineering GmbH) were analyzed with a custom script to assess lens density and compare it to Scheimplug based images. SS-OCT yielded improved lens imaging compared with the Scheimpﬂug device and had a higher correlation with clinical parameters such as the energy required during phacoemulsification surgery [10].

Unlike other AI-based classification models, the model presented here does not require pre-processing of the underlying OCT images, such as demarcating the lens or reducing background noise [5,14]. In addition, only two perpendicular images were used per eye. The algorithm presented here focuses on the lens region and extracts the necessary information to assess lens density in nuclear cataract and PSC from the correct areas of the entire anterior segment OCT image, as shown by the grad-CAM results.

Since PSC encompasses only the subcapsular region of the lens, the area of interest for analysis is much smaller than when focusing on nuclear cataractous changes. PSC’s visually disturbing effect is not based on its density but rather on a light scattering effect. Even more interesting is the effectiveness of the AI-algorithm to discriminate PSC cataracts, since the tested AS-OCT operates at a wavelength of 840 nm, which is longer than that of visible light and therefore scatters less.

Automated classification of cataract severity based on anterior segment OCT images could contribute to greater standardization in both research and daily clinical practice, as well as to significantly reducing time and costs. For example, standardized mass screening for epidemiological studies could be facilitated, or standardized recording of cataract grade in clinical trials could be ensured, with a significant reduction in effort. The AI-based classification model could also personalize laser energy applied during FLACS in everyday clinical practice. Currently, although almost all FLACS platforms are equipped with integrated OCT imaging system [18,19], these are used primarily to ensure accurate localization of laser delivery. The ALLY™ Adaptive Cataract Treatment System (LENSAR) employs Scheimpflug imaging to classify cataracts into five density categories and to identify the lens nucleus, thereby generating recommendations for fragmentation patterns. However, Scheimpflug-based cataract grading has been shown to be inferior to OCT based grading by Mackenbrock et al. [10]. Pupil size can vary after FLACS application [20]. Since GRAD-CAM results demonstrate a focus on the central lens region, this algorithm is applicable with various pupil sizes.

The study presented is accompanied by the usual limitations of a feasibility study. A more comprehensive, evenly distributed base data set, including data from marginal LOCS III NO and PSC grades, would contribute to a more robust result. Since assessment of cataract grades in our model is based on real patient OCT images and mixed forms of cataracts such as nuclear, cortical and PSC cataracts are also represented. (S1 Appendix). This can lead to misinterpretation by the network and decrease specificity and sensitivity. In order to simplify the grading system, we avoided overlapping cataract entities such as cortical and PSC cataracts. Because cortical cataract has little influence on lens hardness, it is therefore less important for adaptation of laser energy during FLACS. In addition, cortical cataract is located in the periphery of the lens which depending on pupil dilation can be hidden behind the iris. Nevertheless, the algorithm’s performance in PSC cataract grading underscores its potential utility in grading cortical cataracts as well, especially when the cortical opacifications are located in the center of the lens and anterior. To confirm this assumption, further studies of all LOCS III types are needed.

In conclusion, although not yet suitable for routine clinical use, initial research using OCT images and deep learning methods for rough classification of cataracts shows promising results. Including more image data, especially LOCS III grades from patients with clear lenses and higher grades, may improve the model’s performance and lead to better classifications.

## Supporting information

S1 Appendix Anonymized data of all LOCS classifications. (XLSX)
